# Growth of Fullerene Fragments Using the Diels-Alder Cycloaddition Reaction: First Step towards a C_60_ Synthesis by Dimerization

**DOI:** 10.3390/molecules18022243

**Published:** 2013-02-13

**Authors:** Martha Mojica, Francisco Méndez, Julio A. Alonso

**Affiliations:** 1 Departamento de Química, División de Ciencias Básicas e Ingeniería, Universidad Autónoma Metropolitana, Unidad Iztapalapa, A.P. 55-534, México, D.F., 09340, Mexico; E-Mail: cbi209382312@xanum.uam.mx; 2 Departamento de Física Teórica, Atómica y Óptica, Facultad de Ciencias, Universidad de Valladolid, E-47011Valladolid, Spain; E-Mail: jaalonso@fta.uva.es

**Keywords:** Diels-Alder cycloaddition, fullerene fragments, transition states, triindenetriphenilene, pentacyclopentacorannulene

## Abstract

Density Functional Theory has been used to model the Diels-Alder reactions of the fullerene fragments triindenetriphenilene and pentacyclopentacorannulene with ethylene and 1,3-butadiene. The purpose is to prove the feasibility of using Diels-Alder cycloaddition reactions to grow fullerene fragments step by step, and to dimerize fullerene fragments, as a way to obtain C_60_. The dienophile character of the fullerene fragments is dominant, and the reaction of butadiene with pentacyclopentacorannulene is favored.

## 1. Introduction

After the discovery of C_60_ by Kroto *et al.* in 1985 [1], its chemical synthesis has been an intensely pursued target. Krätschmer *et al.* [2] developed in 1990 a technique to produce C_60_ based on the vaporization of graphite on a helium atmosphere, and later Scott and coworkers [3] synthesized C_60_ by the pyrolysis of the polycyclic aromatic hydrocarbon precursor C_60_H_27_Cl_3_. However, the chemical synthesis of C_60_ cannot be considered a solved problem [4] due to the inherent disadvantages of both techniques. In the first case, the low yield and the problems of separating and purifying the products make the fullerene synthesis inefficient and expensive [5]. In the second, the low yield problem can be solved by using a Pt catalyst [6], however, the pyrolytic treatment employed makes necessary the creation of new methodologies to synthesize fullerenes.

**Figure 1:**
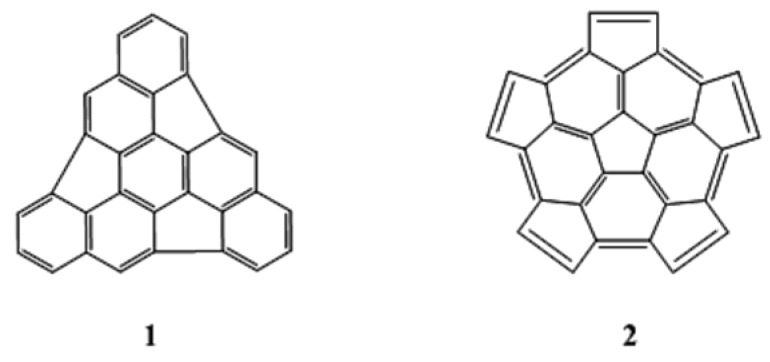
Triindenetriphenilene (**1**) and pentacyclopentacorannulene (**2**).

One particularly seductive and promising strategy is the polymerization of C_60_ fragments, especially the dimerization of two identical hemispherical hydrocarbons, C_30_H_x_ [7,8,9,10,11]. In this case the curvature is already present in the fragments, and the main challenge is to find a way to “stitch” the fragments together to give the closed fullerene. Obviously, the choice of those fragments is a key condition for the success of this methodology. Using “La Coupe du Roi” method [12], Geneste and coworkers [9] found nine isometric C_30_ fragments derived from C_60_, and they determined that the triindenetriphenilene and the pentacyclopentacorannulene fragments (labeled **1** and **2** in Figure 1, respectively) were the most stable fragments. 

Recently, Scott and coworkers [13] have used the Diels-Alder (DA) cycloaddition reaction to synthesize carbon nanotubes in solution, with single chiralities and uniform diameters. Addition of an acetylene dienophile molecule to the bay regions on the rim of a buckybowl (a fragment of a buckyball acting as the end-cap of a nanotube), or an aromatic belt, leads to the nanotube growth by the formation of new six-membered rings [14]. They also calculated the activation energies for the addition of acetylene to different aromatic hydrocarbons and observed that the activation energy barrier decreases as the size of the hydrocarbon molecule increases. Moreover, they proposed the DA reaction of 7,14-dimesitylbisanthene and acetylene to experimentally probe the methodology, and concluded that the reaction was relatively easy under mild conditions. Latter they used a benzyne molecule as masked acetylene to achieve the addition of two rings at once [15]. This evidence suggested us the idea of using the DA cycloaddition reaction in the synthesis of C_60_. In this paper we describe the calculations that we have performed to study the DA cycloaddition reactions between fragments **1** and **2** with ethylene (**e**) and butadiene (**b**) molecules, because of the opportunity of forming new six-membered carbon rings in the rims of the two fragments. The study supports the feasibility of a systematic method to obtain fullerenes by DA cycloadditions to a starting fragment, and also suggests the possibility of assembling fullerenes by dimerization of fragments. 

## 2. Results and Discussion 

**Figure 2:**
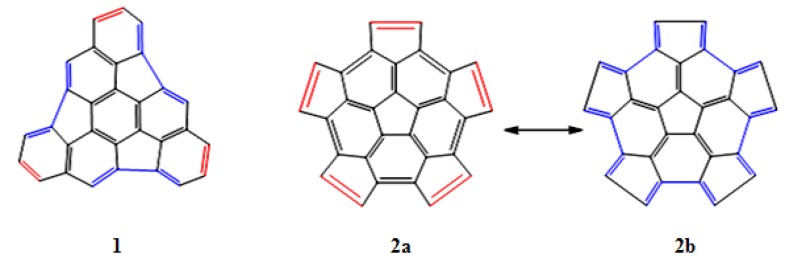
Regions in which the fragments **1** and **2** behave as a diene (blue color), or as a dienophile (red color).

The structures of fragments **1** and **2** were analyzed and the regions that behave as a diene or a dienophile have been identified (see Figure 2). In fragment **1**, these alternating regions are separated by one or two bonds, while in fragment **2** the regions are adjacent and share carbon atoms; this is clearly appreciated in the resonance structures **2a** and **2b**.

**Figure 3:**
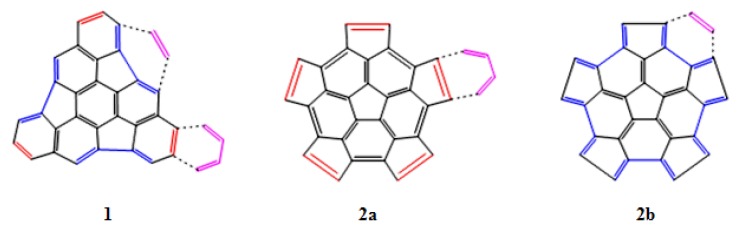
DA reaction of the fragments **1** and **2** with a diene (1,3-butadiene) and a dienophile (ethylene).

Based on the structure of fragments **1** and **2**, we have studied two main reactions. In the first case the DA reaction is carried out with 1,3-butadiene as the diene and the fragments **1** or **2** as the dienophile (Figure 3, panels **1** and **2a**). In the second case, the DA reaction is between ethylene as the dienophile and fragments **1** or **2** as the diene (Figure 3, panels **1** and **2b**). We have also analyzed the preference for the addition on the concave (*endo*) or convex (*exo*) surfaces of the fragments.

### 2.1. Frontier Molecular Orbital Analysis

On the basis of Frontier Molecular Orbitals (FMO) theory [16,17], the Diels-Alder reaction proceeds through the interaction of the Highest Occupied Molecular Orbital (HOMO) of one of the molecules and the Lowest Unoccupied Molecular Orbital (LUMO) of the other molecule. When the interaction is between the HOMO of the diene and the LUMO of the dienophile, the reaction is called Normal Electron Demand Diels-Alder (NEDDA) reaction, and when the interaction is between the HOMO of the dienophile and the LUMO of the diene the reaction is called Inverse Electron Demand Diels-Alder (IEDDA) reaction [18]. As the energy difference between the two involved orbitals (HOMO-LUMO gap) decreases, the reaction should be easier. 

Table 1 reports the energies of the HOMO and LUMO of the **e** and **b** molecules and the fragments **1** and **2**, obtained from the canonical orbitals calculated at the HF/6-31G(d,p)// B3LYP/6-31G(d,p) level of theory. That is, the geometries were optimized at the B3LYP/6-31G(d,p) level and then a calculation at the HF/6-31G(d,p) level was performed to obtain the orbital energies of the canonical orbitals. These orbital energies are in good agreement with the HOMO and LUMO energies calculated by Park *et al.* [19] with the MP2 method [20]. Table 2 shows the HOMO-LUMO gaps for the NEDDA and IEDDA reactions, that is, ΔE_NEDDA_ = E_LUMOdienophile_ − E_HOMOdiene_, and ΔE_IEDDA_ = E_LUMOdiene_ − E_HOMOdienopile_, and also the difference δΔE = ΔE_IEDDA_ − ΔE_NEDDA_. Values of δΔE < 0 indicate that an IEDDA reaction is preferred, and values of δΔE > 0 indicate the preference for a NEDDA reaction. The largest IEDDA and NEDDA HOMO-LUMO gaps occur for the reference reaction between **e** and **b**. The gaps for the reactions between fragments **1** and **2** with **e** and **b** are smaller, and consequently these reactions should be easier. In addition, the reactions of **e** or **b** with fragment **2** are preferred over the corresponding reactions with fragment **1**. Moreover, the reactions of fragments **1** and **2** with **b** are favored over the corresponding reactions of fragments **1** and **2** with **e**. 

**Figure 4:**
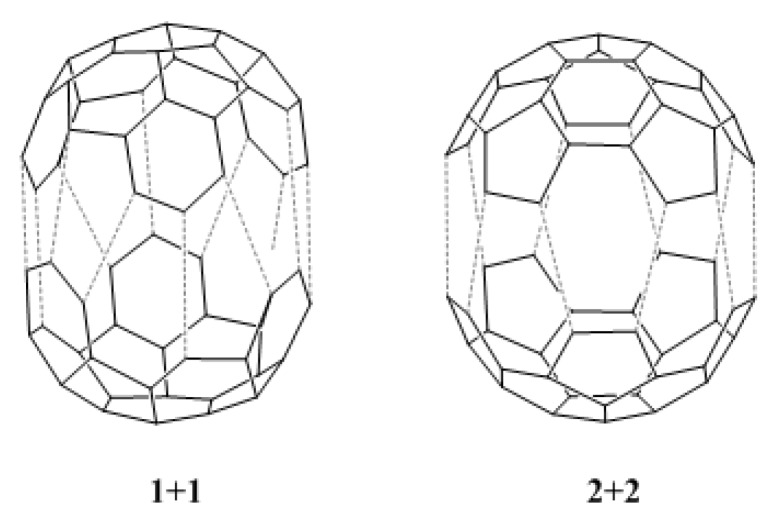
Dimerization reaction between two fragments of type **1** (**1** + **1**), and between two fragments of type **2** (**2** + **2**).

We have also calculated the HOMO-LUMO gaps for the dimerization reaction between two fragments of type **1**, and between two fragments of type **2** (see Figure 4). The values obtained, 8.66 eV and 7.85 eV, respectively, are lower than the values for the reactions shown in Table 2. This suggests that the dimerization reactions **1** + **1** and **2** + **2** should be possible, the dimerization of fragment **2** being preferred over the dimerization of fragment **1**. Moreover, these values indicate that both dimerization reactions should be easier than the reaction between **e** and **b**. However, chemical reactions do not only depend on electronic effects [21], and an analysis of kinetic and thermodynamic factors is required for the complete understanding of the proposed reactions.

### 2.2. Kinetic and Thermodynamic Analysis

**Figure 5:**
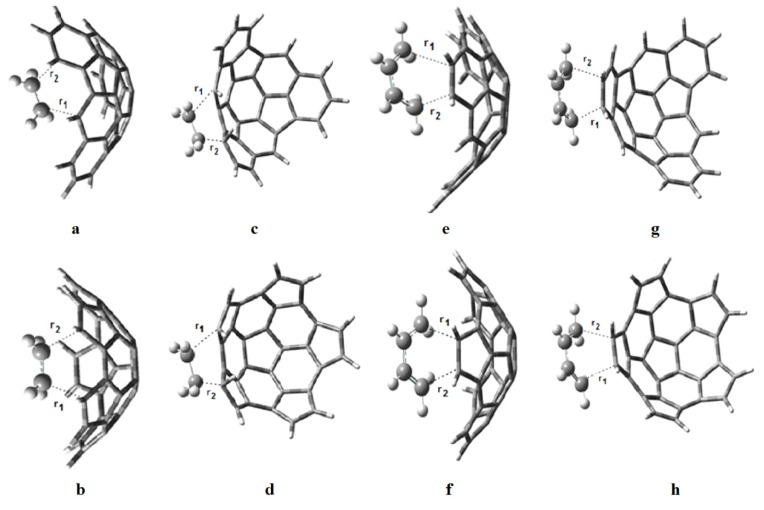
Transition state structures for the reactions of (**a**) **e** onto the concave surface of **1**; (**b**) **e** onto the concave surface of **2**; (**c**) **e** onto the convex surface of **1**; (**d**) **e** onto the convex surface of **2**; (**e**) **b** onto the concave surface of **1**; (**f**) **b** onto the concave surface of **2**; (**g**) **b** onto the convex surface of **1**; and (**h**) **b** onto the convex surface of **2**.

The transition state structures of the proposed reactions are shown in Figure 5, and the transition distances r_1_ and r_2_ are given in Table 3. Small values of Δr = r_1_ − r_2_ suggest synchronous reactions while large values of Δr suggest asynchronous reactions. The reactions of **e** onto the convex surface of fragments **1** and **2** will be the most asynchronous ones, followed by the reactions of **b** onto the concave and convex surfaces of fragment **1**. The reaction of **b** onto the convex surface of fragment **2** will be fully synchronous (Δr = 0), as well as the reaction of **e** and **b**; moreover, the transition distances in both reactions are equal (2.27Å). This value is in good agreement with the values reported by Houk *et al.* [22] for the reaction between **e** and **b**. 

Table 4 summarizes the calculated activation energies *E_a_*, the standard activation Gibbs energies Δ*G_a_*, standard activation enthalpies Δ*H_a_*, and standard activation entropies Δ*S_a_* for the eight transition states of Figure 5, and for the reaction between **e** and **b**. The interactions with ethylene are favored on the concave surface of the fragments, while the reactions with butadiene are preferred on the convex surface. Butadiene, being a larger molecule, gives rise to steric effects that make the reaction over the concave surface difficult, leading thus to a lower activation barrier for the approach onto the convex surface. The calculated activation energy of reaction **e**+**b** is 24.82 kcal mol^−1^. Therefore, reactions with equal or lower activation energies should proceed easily. The reaction of **b** onto the convex surface of fragment **2** shows the lowest activation energy, 22.61 kcal mol^−1^, which means that this reaction would be possible under mild conditions. The activation energy for the same reaction on the concave surface is 8 kcal per mol higher, because of the steric repulsion between **b** and the electron cloud at the concave surface of the fragment. This makes the interaction difficult. All the other reactions have higher activation energies. Therefore, those reactions would require a larger amount of energy to proceed, and this could be translated into higher temperatures and pressures, which make the experimental conditions similar to those employed in the traditional synthesis methods. Considering the reactions of **e** and **b** with fragments **1** and **2**, in general the reactivity trend predicted by FMO is similar to that derived from *E_a_*, except for the relative ordering of the reactions of **e** with fragments **1** and **2**. The reactions in which the fragment **2** acts as diene have higher *E_a_* values compared to reactions in which the fragment **1** acts as diene. Following Scott’s ideas, we have also calculated the transition state for the reaction of fragment **2** with acetylene as the dienophile. The activation energy of this reaction, E_a_ = 40.07 kcal mol^−1^, is close to that of the reaction of fragment **2** with ethylene, and this suggests that the use of acetylene as a dienophile is not adequate for the growth of the fragment.

**Figure 6:**
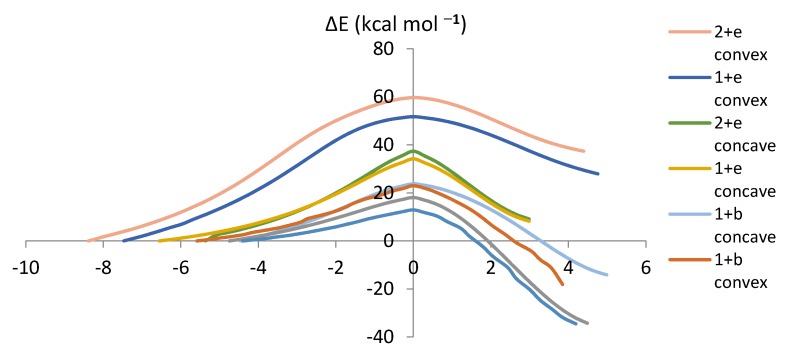
Plots of energy (kcal mol^−1^) *versus* the IRC (in bohr/amu^1/2^) for the proposed reactions.

The Intrinsic Reaction Coordinate (IRC) determines the energy as well as the geometry of the reacting system [25,26]. The energy change along the IRC gives the potential barrier shape [27]. Figure 6 summarizes the IRCs of the proposed reactions. The analysis of the reaction mechanisms shows that the transition state structures (IRC = 0) leads to the DA cycloadducts in the forward direction and the reagents in the reverse direction without any evidence of intermediates or complex structures along the reaction paths (Figure 6). The IRC summarizes the trend predicted by FMO (except for the relative ordering of the reactions of **e** with fragments **1** and **2**) and those derived from *E_a_*.

The thermodynamic parameters for the reactions studied are collected in Table 5. All those reactions are entropically disfavored. The reactions of fragments **1** and **2** with **b** are exothermic (negative Δ*H_r_*), while the reactions with **e** are endothermic (positive Δ*H_r_*). Only the reference reaction **e**+**b** and the reactions between fragment **2** and **b** are spontaneous, having negative values of Δ*G_r_*. These reactions indicate the dienophilic character of fragment **2**. This result reinforces the possibility of synthesizing larger fullerene fragments starting with fragment **2**, and it also suggests the dimerization of fragment **2**, that is, the reaction **2**+**2**, as a promising method to produce C_60_. 

**Figure 7:**
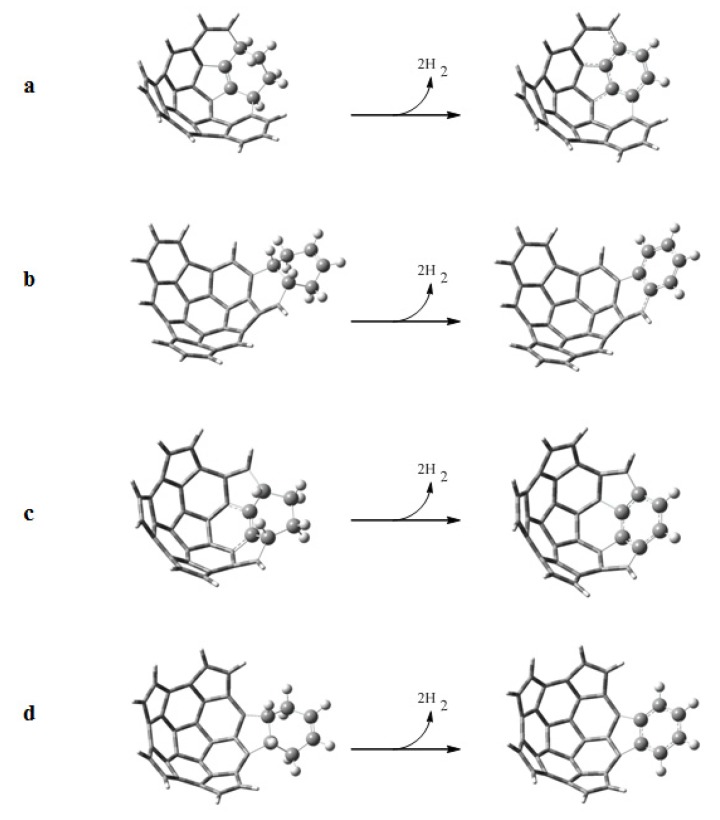
Dehydrogenation reactions to aromatize the adducts obtained from the Diels-Alder reactions of a) **1** + **e**, b) **1** + **b**, c) **2** + **e** and d) **2** + **b**.

Following the scheme proposed by Scott, we have studied the dehydrogenation reactions of the adducts formed by the Diels-Alder reactions. Dehydrogenation leads to the aromatization of the adducts. The dehydrogenation reactions are shown in Figure 7, and the standard Gibbs energies Δ*G_r_*, enthalpies Δ*H_r_*, and entropies *T*Δ*S_r_* of reaction are given in Table 6. We observe that the reactions with fragment **2** are endothermic (positive Δ*H_r_*), except for the reaction with **e** onto the convex surface. On the other hand the reactions with fragment **1** are exothermic, with the exception of the reaction with **e** onto the concave surface. However, all the reactions are spontaneous (negative Δ*G_r_*) because of the strongly favorable entropy contribution (*T*Δ*S_r_* ≈ 17 kcal mol^−1^). The values of Δ*G_r_* for the aromatization of the adducts formed in the reactions between **e** and fragments **1** and **2** over the convex surface are more negative because of the lower stability of those adducts.

## 3. Experimental

The calculations described in this work have been carried out with the Gaussian 03 (G03) program package [29]. The geometries were fully optimized at the B3LYP/6-31G(d,p) level of theory. This method and basis set have been widely used in the study of fullerene reactivity [30,31], leading to good agreement with geometrical parameters of fullerene fragments obtained by X-ray diffraction [32]. The methodology has proven to describe satisfactorily the DA cycloaddition reactions over fullerenes and fullerene fragments [33,34]. Therefore, the kinetic and thermodynamic parameters were calculated at the B3LYP/6-31G(d,p) level of theory as well. The transition states (TS) were located with the QST2 G03 optimization option of the code; for all of them, vibrational frequency analyses were carried out. A single imaginary frequency was located for each transition state. All frequencies are real for the minima. The electronic energies of minima and transition states were corrected by the inclusion of zero-point energies. The Intrinsic Reaction Coordinates (IRCs) were determined from the corresponding TS using the IRC G03 keyword. In each case the FORWARD and REVERSE sections were calculated independently.

## 4. Conclusions

The calculations performed show the feasibility of the Diels-Alder reaction as a method to increase the size of fullerene fragments step by step. We have studied the Diels-Alder reactions of ethylene and butadiene with two fragments, triindenetriphenilene (Fragment **1**) and pentacyclopentacorannulene (Fragment **2**). The Frontier Molecular Orbitals analysis indicates that the reactions in which the fullerene fragments behave as a dienophile are preferred, and this was confirmed by the kinetic and thermodynamic analysis. The transition states indicate that the interactions with ethylene are favored on the concave surface of the fragments, while the reactions with butadiene, a larger molecule, are preferred on the convex surface. 

Even if fragment **1** is more stable than fragment **2** owing to its lower pyramidalization of the bonds, the most favorable addition reactions are those of butadiene with fragment **2**. In this particular case, the activation barriers are lower than for other reactions, and in addition, the free energies of reaction are negative, in contrast with the positive values for other reactions. The negative values of the free energy for the dehydrogenation reactions of the adducts favor the growth of the fragments. Motivated by these results and others [35,36], experimental studies of the reactivity of the current systems are underway.

## Figures and Tables

**Table 1:** HOMO and LUMO energies (in eV) of ethylene, 1,3-butadiene and the fragments **1** and **2**.

Molecule	E_HOMO_	E_LUMO_
Ethylene	−10.11 (−10.07) ^a^	4.88 (4.87) ^a^
Butadiene	−8.77 (−8.82) ^a^	3.45 (3.59) ^a^
1	−7.28	1.38
2	−7.18	0.67

^a^ from ref. [19].

**Table 2:** HOMO-LUMO gaps, in eV, for the NEDDA and IEDDA reactions of fragments **1** and **2** with ethylene and 1,3-butadiene. ΔE_NEDDA_ = E_LUMOdienophile_ − E_HOMOdiene_ and ΔE_IEDDA_ = E_LUMOdiene_ − E_HOMOdienopile_. Also, δΔE = ΔE_IEDDA_ − ΔE_NEDDA_. Results for reaction **e** + **b** are also included.

Reaction	ΔE_NEDDA_	ΔE_IEDDA_	δΔE
e + b	13.66 (13.69) ^a^	13.56 (13.66) ^a^	−0.09
1 + e	12.17	11.49	−0.67
2 + e	12.06	10.78	−1.27
1 + b	10.15	10.73	0.58
2 + b	9.45	10.63	1.18

^a^ from ref. [19].

**Table 3:** Distances r_1_ and r_2_, in Å, for the transition states shown in Figure 5. Also Δr = r_1_ − r_2_.

TS	r_1_	r_2_	Δr
e + b	2.27 (2.21) ^a^	2.27 (2.21) ^a^	0
1 + e concave	2.09	2.07	0.02
2 + e concave	2.07	2.06	0.01
1 + e convex	2.41	1.68	0.73
2 + e convex	2.31	1.63	0.67
1 + b concave	2.49	1.92	0.58
2 + b concave	2.28	2.25	0.03
1 + b convex	2.36	2.02	0.34
2 + b convex	2.27	2.27	0

^a^ from reference [22].

**Table 4:** Activation energy *E_a_*, standard activation Gibbs energy Δ*G_a_*, standard activation enthalpy Δ*H_a_*, and standard activation entropy *T*Δ*S_a_* for Diels-Alder reactions of **e** and **b** with fragments **1** and **2**. Data for the Diels-Alder reaction between **e** and **b** is given as reference.

Reaction	*E_a_* (kcal mol^−1^)	Δ*G_a_* (kcal mol^−1^)	Δ*H_a_* (kcal mol^−1^)	*T*Δ*S_a_* (kcal mol^−1^)
e + b	24.8 (27.5) ^a^ (24.2–26.7)^b^ (25.9) ^c^	36.35	23.45 (24.2)^d^ (24.9)^e^	−12.91 (−13.32)^f^ (−12.76)^g^
1 + e concave	40.71	52.88	39.62	−13.25
2 + e concave	45.63	57.21	44.70	−12.51
1 + e convex	67.89	80.22	66.75	−13.47
2 + e convex	73.54	85.84	72.35	−13.49
1 + b concave	39.01	51.86	38.36	−13.49
2 + b concave	31.05	43.53	30.43	−13.10
1 + b convex	33.11	46.03	32.47	−13.56
2 + b convex	22.61	35.52	22.02	−13.51

^a^ Experimental *E_a_* from ref. [23]; ^b^ Experimental values from *E_a_* of cyclohexene cycloreversion and the experimental heat of reaction [23]; ^c^ Calculated *E_a_* from ref. [23]; ^d^ Experimental Δ*H_a_* from ref. [24]; ^e^ Calculated Δ*H_a_* from ref. [24]; ^f^ Experimental value of *T*Δ*S_a_* from ref. [24], ^g^ Calculated value of *T*Δ*S_a_* from ref. [24].

**Table 5:** Standard Gibbs energies Δ*G_r_*, standard enthalpies Δ*H_r_*, and standard entropies *T*Δ*S_r_* of reaction.

Reaction	Δ*G_r_* (kcal mol^−1^)	Δ*H_r_* (kcal mol^−1^)	*T*Δ*S_r_* (kcal mol^−1^)
e + b	−23.76	−37.82 (−37.9) ^a^ (−36.6) ^b^	−14.06 (−14.1)^c^
1 + e concave	16.03	1.73	−14.29
2 + e concave	21.90	7.86	−14.04
1 + e convex	49.53	35.36	−14.17
2 + e convex	60.84	46.82	−14.02
1 + b concave	6.62	−8.49	−15.11
2 + b concave	−10.41	−25.50	−15.09
1 + b convex	10.34	−4.28	−14.62
2 + b convex	−13.05	−27.88	−14.82

^a^ Experimental Δ*H_r_* from reference [24]; ^b^ Calculated Δ*H_r_* from reference [24]; ^c^ Calculated value of *T*Δ*S_r_* from reference [24].

**Table 6:** Standard Gibbs energy Δ*G_r_*, standard enthalpy Δ*H_r_*, and standard entropy *T*Δ*S_r_* of the dehydrogenation reaction.

System	ΔG*_r_*(kcal mol^−1^)	ΔH*_r_* (kcal mol^−1^)	TΔS_r_ (kcal mol^−1^)
e + b	0.09	17.20 (21.13) ^a^	17.12
1 + e concave	−11.34	5.93	17.27
2 + e concave	−12.23	4.96	17.19
1 + e convex	−44.85	−27.70	17.15
2 + e convex	−51.16	−33.99	17.17
1 + b concave	−22.84	−5.58	17.26
2 + b concave	−13.12	4.14	17.25
1 + b convex	−26.56	−9.80	16.77
2 + b convex	−10.48	6.51	16.99

^a^ Experimental value of Δ*H_r_* value obtained from the hydrogenation reaction [28].
